# The effectiveness of influenza vaccination in preventing hospitalisations of elderly individuals in two influenza seasons: a multicentre case–control study, Spain, 2013/14 and 2014/15

**DOI:** 10.2807/1560-7917.ES.2017.22.34.30602

**Published:** 2017-08-24

**Authors:** Angela Domínguez, Núria Soldevila, Diana Toledo, Pere Godoy, Elena Espejo, Maria Amelia Fernandez, José María Mayoral, Jesús Castilla, Mikel Egurrola, Sonia Tamames, Jenaro Astray, María Morales-Suárez-Varela, the Working Group of the Project PI12/02079

**Affiliations:** 1 Departament de Medicina, Universitat de Barcelona, Barcelona, Spain; 2 CIBER Epidemiología y Salud Pública (CIBERESP), Madrid, Spain; 3 Agència de Salut Pública de Catalunya, Barcelona, Spain; 4 Institut de Recerca Biomèdica de Lleida, Universitat de Lleida, Lleida, Spain; 5 Hospital de Terrassa, Terrassa, Spain; 6 Complejo Hospitalario Universitario de Granada, Granada, Spain; 7 Servicio de Vigilancia de Andalucía, Sevilla, Spain; 8 Instituto de Salud Pública de Navarra (IdiSNA), Pamplona, Spain; 9 Hospital de Galdakao, Usansolo, Spain; 10 Dirección General de Salud Pública, Investigación, Desarrollo e Innovación, Junta de Castilla y León, León, Spain; 11 Consejería de Sanidad, Madrid, Spain; 12 Departamento de Medicina Preventiva, Universidad de Valencia, Valencia, Spain

**Keywords:** effectiveness, elderly, influenza, matched case–control, vaccination, hospitalised cases, hospitalised controls

## Abstract

Influenza vaccination may limit the impact of influenza in the community. The aim of this study was to assess the effectiveness of influenza vaccination in preventing hospitalisation in individuals aged ≥ 65 years in Spain. A multicentre case–control study was conducted in 20 Spanish hospitals during 2013/14 and 2014/15. Patients aged ≥ 65 years who were hospitalised with laboratory-confirmed influenza were matched with controls according to sex, age and date of hospitalisation. Adjusted vaccine effectiveness (VE) was calculated by multivariate conditional logistic regression. A total of 728 cases and 1,826 matched controls were included in the study. Overall VE was 36% (95% confidence interval (CI): 22–47). VE was 51% (95% CI: 15–71) in patients without high-risk medical conditions and 30% (95% CI: 14–44) in patients with them. VE was 39% (95% CI: 20–53) in patients aged 65–79 years and 34% (95% CI: 11–51) in patients aged ≥ 80 years, and was greater against the influenza A(H1N1)pdm09 subtype than the A(H3N2) subtype. Influenza vaccination was effective in preventing hospitalisations of elderly individuals.

## Introduction

Influenza is an acute illness caused by influenza viruses. During seasonal epidemics, large numbers of influenza infections occur in all age groups. In most individuals, influenza is a self-limiting illness, but serious secondary complications appear in some of those infected with the influenza viruses. Influenza virus infection-related morbidity and mortality is a serious human health concern worldwide, affecting health of populations and economies worldwide. The illness may result in hospitalisation, overwhelming hospitals and causing excess influenza health-related deaths [1]. Worldwide, annual epidemics are estimated to result in ca 3 to 5 million cases of severe illness and ca 250,000 to 500,000 deaths [2]. Individuals who are elderly, especially those with comorbidities, are particularly at risk for influenza-related complications and frequently require hospitalisation. In an American study carried out in the 2005/06 through 2013/14 seasons, 89% of all influenza-associated deaths were in people aged ≥ 65 years [3]. A recent French study estimated that 11% of all-cause deaths in elderly individuals during the influenza season were attributable to influenza [4]. However, mortality is just the tip of the iceberg in terms of disease and the economic burden, and hospitalisation is also an important outcome that should be considered [5].

The capacity of influenza viruses to undergo gradual antigenic change in their surface antigens is a challenge for vaccination against seasonal influenza. Annual administration of the seasonal influenza vaccine, especially in those known to be at high risk of serious complications as a result of influenza, is the focus of current efforts to reduce the disease impact [1]. In the 2013/14 season, the trivalent inactivated vaccine administered in Spain and in all the northern hemisphere, contained an A/California/7/2009(H1N1)pdm-09-like virus, an A(H3N2) virus antigenically similar to the cell-propagated prototype virus A/Victoria/361/2011 and a B/Massachusetts/2/2011-like virus. For the 2014/15 season, the vaccine composition only changed the A/Victoria/361/2011 component to the A/Texas/50/2012 component, an antigenically similar virus.

Various factors affect influenza vaccine effectiveness (VE). One main factor is the antigenic similarity or dissimilarity between circulating strains and vaccine strains: VE decreases with increasing antigenic distance between vaccine components and circulating strains [6]. There was no mismatch in 2013/14 for the A(H1N1)pdm09 and A(H3N2) components but in 2014/15, some degree of mismatch for the A(H3N2) circulating strain was observed [7,8]. Another factor is the influenza illness rate, which may vary substantially from year to year; in years with low rates, the power of some studies to detect significant VE may be compromised [9]. Therefore, studies including more than one season are recommended in order to estimate VE.

The aim of this study was to assess the effectiveness of influenza vaccination in preventing hospitalisation due to laboratory-confirmed influenza in individuals aged ≥ 65 years during two influenza seasons (2013/14 and 2014/15) in Spain.

## Methods

### Study design

We carried out a multicentre case–control study in 20 major hospitals from seven of 17 Spanish regions (Andalusia, the Basque Country, Catalonia, Castile and Leon, Madrid, Navarra and Valencian Community), covering 1,444,688 individuals aged ≥ 65 years and representing 16.8% of the Spanish population in this age group. Cases and corresponding controls admitted to participating hospitals between December 2013 and March 2015 were recruited.

### Selection of cases and controls

We selected patients aged ≥ 65 years who were hospitalised for at least 24 hours with laboratory-confirmed (PCR, culture or immunofluorescence) influenza virus infection.

For each case, up to three matched controls from among patients aged ≥ 65 years with unplanned hospital admission due to causes other than influenza or acute respiratory disease were selected. Controls were matched with each case according to sex, age (± 3 years) and date of hospitalisation (± 10 days). They were selected from patients admitted to the internal medicine, general surgery, otorhinolaryngology, ophthalmology, dermatology or traumatology services wards. Patients referred from nursing homes and those who did not provide written informed consent were excluded.

### Data collection

The following demographic variables and pre-existing medical conditions were recorded: age, sex, marital status, educational level, smoking and high alcohol consumption (> 40 g/day for men and > 24 g/day for women), number of hospital visits during the last year, the Barthel Index as a measurement of limitations in activity (ranging from 0 (complete dependence) to 100 (complete independence)), chronic obstructive pulmonary disease (COPD), chronic respiratory failure, history of pneumonia during the last two years, other lung diseases, neoplasia, transplantation, immunosuppressive treatment, asplenia, diabetes mellitus, renal failure, nephrotic syndrome, autoimmune disease, AIDS, asymptomatic HIV infection, congestive heart disease, disabling neurological disease, obesity (body mass index ≥ 30 kg/m^2^), chronic liver disease, haemoglobinopathy or anaemia, cognitive dysfunction, convulsions and neuromuscular disease. Information on influenza vaccination in the current and previous season, and information on pneumococcal vaccination was collected.

Cases were considered vaccinated with the current influenza vaccine or pneumococcal vaccine if they had received a dose of the vaccine ≥ 14 days before symptom onset. Controls were considered vaccinated if they had received a dose of the influenza vaccine ≥ 14 days before the onset of symptoms of the matched case. Influenza vaccination in the previous season in cases and controls was defined as administration of the seasonal influenza vaccine during the preceding influenza season.

### Statistical analysis

A bivariate comparison for matched data of demographic variables and medical conditions between cases and controls was made using McNemar’s test. A two-tailed distribution was assumed for all p values.

A univariate conditional logistic regression model was used to estimate the crude VE in preventing influenza hospitalisation. Propensity score (PS) analysis was used to evaluate the adjusted VE. The PS was created using a logistic regression model with influenza vaccination status as the outcome and demographic variables, medical conditions and functional status as independent variables. The PS was used as a continuous covariate in a final conditional logistic regression model.

Using the formula *VE = (1 − OR) × 100*, VE was calculated globally, by season, for the presence of high-risk medical conditions, for age groups, for type/subtype of influenza virus and for each category of vaccine exposure: vaccinated only in current season, only in prior season, in both seasons, and unvaccinated in both seasons as the reference group.

The analysis was performed using the SPSS version 23 statistical package and the R version 3.3.0 statistical software [10].

### Ethical considerations

All data collected were treated as confidential, in strict observance of legislation on observational studies. The study was approved by the ethics committees of the participating hospitals. Written informed consent was obtained from all patients included in the study.

## Results

A total of 728 cases and 1,826 controls were included in the study. The distribution of cases and controls according to demographic variables, medical conditions and vaccination history is shown in Table 1. A total of 359 cases (49.3%) and 1,053 controls (57.7%) had received influenza vaccination. Of the 728 cases, 433 were from the 2013/14 season and 295 were from the 2014/15 season. Of the 433 cases from the 2013/14 season, 429 (99.1%) were infected with influenza A virus (59.8% were A(H1N1)pdm09, 30.5% were A(H3N2) and 8.8% were unsubtyped), two cases were infected with influenza B virus and two cases were missing data for type and subtype. Of the 295 cases from the 2014/15 season, 258 (87.5%) were infected with influenza A virus (22.4% were A(H1N1)pdm09, 42.0% were A(H3N2) and 23.1% were unsubtyped) and 37 (12.5%) were infected with influenza B virus.

**Table 1:** Distribution of influenza cases and controls aged ≥ 65 years according to demographic variables, medical conditions and history of vaccination, Spain, influenza seasons 2013/14 and 2014/15

Characteristics	Cases (n = 728)		Controls (n = 1,826)		Crude OR	95% CI	p value
	n	%	n	%			
**Age group**							
65–79 years	411	56.5	1,016	57.0	Ref	Ref	0.50
≥ 80 years	317	43.5	810	43.0	0.89	0.64–1.24	
**Sex**							
Female	343	47.1	884	48.4	NA	NA	NA
Male	385	52.9	942	51.6	NA	NA	NA
**Marital status**							
Married/cohabiting	450	61.9	1,020	56.0	Ref	Ref	Ref
Single	39	5.4	145	8.0	0.57	0.39–0.83	0.004
Widowed	217	29.8	615	33.8	0.76	0.61–0.95	0.02
Separated/divorced	21	2.9	42	2.3	1.17	0.68–2.00	0.57
**Educational level**							
Without or primary	560	77.0	1,349	74.9	Ref	Ref	0.07
Secondary or higher	167	23.0	453	25.1	0.81	0.64–1.01	
**Barthel Index^a^**							
0–90**^a^**	276	37.9	796	43.6	0.79	0.64–0.96	0.02
> 90**^a^**	452	62.1	1,028	56.4	Ref	Ref	
**Smoking status**							
No smoker	383	52.6	1,057	57.9	Ref	Ref	0.01
Smoker/ex-smoker	345	47.4	769	42.1	1.39	1.09–1.77	
**High alcohol consumption^b^**							
Yes	16	2.2	53	2.9	0.77	0.43–1.38	0.38
No	712	97.8	1,772	97.1	Ref	Ref	
**Number of hospital visits during the past year**							
0–2	403	56.1	916	50.6	Ref	Ref	0.05
≥ 3	316	43.9	896	49.4	0.82	0.67–1.00	
**High-risk medical conditions**							
No	104	14.3	386	21.1	Ref	Ref	< 0.001
Yes	624	85.7	1,440	78.9	1.73	1.35–2.22	
**Current-season influenza vaccine received**							
Yes	359	49.3	1,053	57.7	0.73	0.61–0.87	< 0.001
No	369	50.7	773	42.3	Ref	Ref	
**Previous-season influenza vaccine received**							
Yes	376	51.6	1,054	57.7	0.78	0.66–0.93	0.005
No	352	48.4	772	42.3	Ref	Ref	
**Pneumococcal vaccine received**							
Yes	372	51.1	836	45.8	1.20	0.99–1.46	0.06
No	356	48.9	990	54.2	Ref	Ref	

CI: confidence interval; NA: not applicable; OR: odds ratio; Ref: reference group for comparison.

^a^ The Barthel Index is a measurement of limitations in activity, ranging from 0 (complete dependence) to 100 (complete independence).

**^b^** High alcohol consumption defined as > 40 g/day for men and > 24 g/day for women.

Most cases (85.7%) and controls (78.9%) had high-risk medical conditions (Table 2).

**Table 2:** Distribution of influenza cases and controls aged ≥ 65 years according to comorbidities, Spain, influenza seasons 2013/14 and 2014/15

Characteristics	Cases (n = 728)		Controls (n = 1,826)		Crude OR	95% CI	p value
	n	%	n	%			
**High-risk medical conditions**							
COPD	194	26.6	218	11.9	3.03	2.37–3.88	< 0.001
Chronic respiratory failure	119	16.3	208	11.4	1.64	1.26–2.14	< 0.001
Pneumonia past 2 years	91	12.5	104	5.7	2.40	1.77–3.26	< 0.001
Other lung disease	238	32.7	380	20.8	1.88	1.54–2.30	< 0.001
Cardiovascular disease	224	30.8	651	35.7	0.84	0.68–1.03	0.09
Diabetes mellitus	235	32.3	666	36.5	0.89	0.74–1.07	0.21
Renal failure with hemodialysis	16	2.2	31	1.7	1.27	0.68–2.37	0.46
Hemaglobinopathy or anaemia	89	12.2	306	16.8	0.68	0.52–0.88	0.004
AIDS	1	0.1	2	0.1	1.30	0.12–14.51	0.83
Asymptomatic HIV infection	3	0.4	1	0.1	9.00	0.94–86.52	0.06
Neurological disease	51	7.0	136	7.4	0.93	0.64–1.33	0.93
Obesity (BMI ≥ 30 kg/m^2^)	174	23.9	370	20.3	1.25	1.01–1.56	0.04
**Non high-risk medical conditions**							
Solid organ neoplasia	103	14.1	348	19.1	0.67	0.52–0.85	0.001
Haematologic neoplasia	39	5.4	40	2.2	2.57	1.62–4.07	< 0.001
Transplantation	22	3.0	10	0.5	5.52	2.52–12.09	< 0.001
Immunosuppressive treatment	35	4.8	67	3.7	1.35	0.87–2.08	0.18
Oral corticosteroid therapy	44	6.0	43	2.4	2.54	1.62–3.97	< 0.001
Asplenia	2	0.3	8	0.4	0.66	0.14–3.11	0.60
Renal failure without hemodialysis	135	18.5	341	18.7	1.01	0.80–1.26	0.94
Nephrotic syndrome	7	1.0	14	0.8	1.14	0.45–2.88	0.78
Autoimmune disease	47	6.5	96	5.3	1.38	0.94–2.03	0.10
Chronic liver disease	28	3.8	95	5.2	0.76	0.49–1.18	0.22
Cognitive dysfunction	76	10.4	205	11.2	0.92	0.68–1.23	0.92
Neuromuscular disease	24	3.3	53	2.9	1.16	0.70–1.93	0.55
Convulsions	8	1.1	23	1.3	0.84	0.37–1.92	0.69

BMI: body mass index; CI: confidence interval; COPD: chronic obstructive pulmonary disease; OR: odds ratio.

The overall adjusted VE against influenza hospitalisation in individuals aged ≥ 65 years was 36% (95% CI: 22–47), without relevant differences between seasons (34%, 95% CI: 10–52 in 2013/14 and 37%, 95% CI: 19–51 in 2014/15) (Table 3). The adjusted VE was greater, but not significantly different, in patients without high-risk medical conditions (51%, 95% CI: 15–71) and in patients aged 65–79 years (39%, 95% CI: 20–53). Adjusted VE was 37% (95% CI: 23–48) for all influenza A viruses, 49% (95% CI: 32–62) for influenza A(H1N1)pdm09 and 26% (95% CI: −3 to 47) for influenza A(H3N2). Protection against influenza B was lower (VE 18%, 95% CI: −145 to 73), but the number of cases was very low (statistical power: 10%).

**Table 3:** Crude and adjusted influenza vaccine effectiveness against hospitalisation because of influenza in individuals aged ≥ 65 years according to influenza season, presence or absence of high-risk medical conditions, case age and type/subtype of influenza virus, Spain, influenza seasons 2013/14 and 2014/15

Variables	Cases vaccinated/n	%	Controls vaccinated/n	%	Crude vaccine effectiveness	95% CI	p value	Adjusted vaccine effectiveness	95% CI	p value
**All**	359/728	49.3	1,053/1,826	57.7	27%	13–39	< 0.001	36%	22–47	< 0.001
**2013/14 season**	208/433	48.0	602/1,038	58.0	31%	13–45	0.002	37%	19–51	< 0.001
**2014/15 season**	151/295	51.2	451/788	57.2	21%	−3 to 40	0.08	34%	10–52	0.01
**Non high-risk medical conditions**	42/104	40.4	159/255	62.4	54%	27–71	0.001	51%	15–71	0.01
**High-risk medical conditions**	317/624	50.8	894/1,571	56.9	21%	7–32	0.01	30%	14–44	< 0.001
**65–79 years of age**	183/411	44.5	561/1,040	53.9	29%	11–44	0.003	39%	20–53	< 0.001
**≥ 80 years of age**	176/317	55.5	492/786	62.6	24%	0–42	0.05	34%	12–51	0.01
**Influenza A**	334/687	48.6	991/1,717	57.7	30%	16–41	< 0.001	37%	23–48	< 0.001
**Influenza A(H1N1)pdm09**	139/325	42.8	464/823	56.4	41%	24–55	< 0.001	49%	32–62	< 0.001
**Influenza A(H3N2)**	138/256	53.9	393/652	60.3	22%	−5 to 42	0.10	26%	−3 to 47	0.08^a^
**Influenza B**	24/39	61.5	58/103	56.3	−35%	−187 to 36	0.43	18%	−145 to 73	0.72^b^

CI: confidence interval

^a^ Statistical power: 74%.

^b^ Statistical power: 10%.

Adjusted VE against hospitalisation was 41% (95% CI: 16–59) among those only vaccinated in the current season and 42% (95% CI: 28–54) among those vaccinated in both the current and previous season. VE among those only vaccinated in the previous season only was 24% (95% CI: −6 to 45) (Table 4).

**Table 4:** Crude and adjusted influenza vaccine effectiveness against hospitalisation because of influenza in individuals aged ≥ 65 years according to current and previous influenza vaccination, Spain, influenza seasons 2013/14 and 2014/15

Vaccination status	Cases (n = 728)		Controls (n = 1,826)		Crude vaccine effectiveness	95% CI	p value	Adjusted vaccine effectiveness	95% CI	p value
	n	%	n	%						
**Vaccinated in current season only**	52	7.1	160	8.8	35%	7–54	0.02	41%	16–59	0.004
**Vaccinated in previous season only**	69	9.5	161	8.8	13%	−20 to 37	0.39	24%	−6 to 45	0.11^a^
**Vaccinated in both seasons**	307	42.2	893	48.9	28%	13–41	0.001	42%	28–54	< 0.001
**Not vaccinated**	300	41.2	612	33.5	Ref	Ref	Ref	Ref	Ref	Ref

CI: confidence interval; Ref: reference group for comparison.

^a^ Statistical power: 54%.

## Discussion

The results of this study over two seasons, one with predominant circulation of influenza A (H1N1)pdm09 and one with A(H3N2) predominance, show overall VE against hospitalisation in individuals aged ≥ 65 years was 36% (95% CI: 22–47).

Some studies investigating the prevention of influenza hospitalisation among individuals who are elderly show greater VE [11,12]. In a German study using the screening method, VE in preventing confirmed influenza hospitalisation in individuals aged ≥ 60 years varied between 62% in the 2011/12 season, when the predominant influenza virus strain was A(H3N2), and 83% in the 2010/11 season, when the predominant strain was A(H1N1)pdm09 [11]. However, these levels of VE might be an overestimate because information on comorbidities was not available to adjust them by [13]. A Spanish case–control study for the 2014/15 season, when the predominant strain was A(H3N2), using test-negative controls in 10 hospitals not included in the present study found a VE of 40% (95% CI: 13–59) in terms of preventing hospital admissions in patients 65 years of age and older [14]. A 2014 New Zealand study, also using a test-negative control design, found a VE of 21% (95% CI: −82 to 66) for influenza-related hospitalisation in patients aged ≥ 65 years [15].

An American test-negative study by Petrie et al. [16] during the 2014/15 season found a VE of 48% (95% CI: −33 to 80) in people aged ≥ 65 years, but the number of individuals included was lower than in the present study. A Chinese test-negative study in people aged > 60 years during the two seasons included in our study, but with a lower number of individuals than in our study, found a point estimate of VE of 27% (95% CI: −114 to 75) during the 2013/14 season. However, no effectiveness was observed in the 2014/15 season [17].

The possible influence of increasing age on VE has been investigated. In our study, adjusted VE against hospitalisations was 39% (95% CI: 20–53) in patients aged 65–79 years and 34% (95% CI: 12–51) in patients aged ≥ 80 years. Decreasing effectiveness has been linked to advanced age in different studies [12,18,19]. Senescence diminishes immunity to influenza infections and the response to vaccination, possibly explaining the lower VE in elderly individuals than in the general population [20].

In terms of analysing VE in older age groups, the German study by Remschmidt et al. found that the VE point estimate against laboratory-confirmed influenza was greater in individuals aged 60–69 years than in older individuals in the 2011/12 season, but the opposite was observed in the 2010/11 season [11]. More research is needed to assess this matter.

In our study, VE was 30% (95% CI: 14–44) in patients with high-risk medical conditions, which was lower than that found in patients without these conditions. Similar results were obtained by other studies [16,21]. In contrast, a 2014/15 Canadian test-negative case–control study of individuals aged ≥1 year by Skowronski et al. [22] did not find a lower age-adjusted VE in patients with comorbidities (16%, 95% CI: −28 to 44) than in patients without comorbidities (6%, 95% CI: −20 to 27). Comorbidities, like age, are strongly associated with a lack of response to vaccination [23]. In fact, one of the major mechanisms through which vaccination is thought to reduce mortality is by blunting influenza-triggered exacerbations of underlying diseases [9]. However, despite the limited VE, the benefits of vaccination may be greater in patients with comorbidities because influenza is associated with a higher risk of severe disease and death in these individuals [24].

Similar to the results of other studies of VE in elderly individuals [11,25], the present study found that VE for subtype A(H1N1)pdm09 was greater (49%, 95% CI: 32–62) than that for subtype A(H3N2) (26%, 95% CI: −3 to 47).

Small, non-significant VE differences were found according to season. In the 2013/14 season, an antigenic mismatch was observed in the B virus component but the A(H1N1)pdm09 and A(H3N2) strains circulating were analogous to the seasonal vaccine strains [7]. However, in some Spanish regions, specific mutations of A(H1N1) and A(H3N2) strains associated with low VE and outbreaks in institutions were found [26]. In the 2014/15 season, mismatched A(H3N2) strains circulated widely around the world [27], but only accounted for 60% of influenza A virus isolates in Spain [8]. This might explain why no relevant differences were found in VE in these two influenza seasons.

In our study, VE in individuals vaccinated only in the current season was similar to that of individuals vaccinated in both the current and previous seasons (41%, 95% CI: 16–59 and 42%, 95% CI: 28–54, respectively), which does not support interference between current and previous vaccination. Three 2014/15 influenza season studies carried out on populations of various ages [16,22,28] reported that vaccination in the previous and current season may diminish VE only in the current season, suggesting negative interference from prior vaccination when the antigenic distance between the vaccine and circulating strains is large but the antigenic distance between vaccine components in consecutive seasons is small [22]. The effects of the various combinations of agent-host factors involved in this phenomenon remain unclear and more research is required to determine their influence on vaccine-induced influenza virus immunity in elderly individuals. However, in agreement with Neuzil [29], we consider that the current policy of administering the influenza vaccine every year should be maintained in the meantime. As the most-vulnerable elderly individuals are those with the most advanced age because they have a higher risk of hospitalisation and death compared with healthy elderly individuals aged 65–75 years [20], seasonal influenza vaccination programs in all elderly individuals should be reinforced.

This study has strengths and limitations. Strengths of this study are the matching design, the high number of covariates recorded and the fact that the vaccination status was obtained by consulting hospital records, vaccination cards and primary health registers.

The limitations include the fact that controls were not systematically swabbed and therefore they may, theoretically, have been infected with influenza virus. However, controls were patients with unplanned admission to hospital because of causes other than influenza or acute respiratory disease, and it seems unlikely that selection bias could invalidate our results. A possible confounder is the functional status; however, we included the Barthel Index in the propensity score and therefore this limitation is reasonably controlled for. Likewise, it is important to consider the weeks with influenza activity in the analysis, but because cases and controls were matched by admission date, we believe this is unlikely to invalidate the results. Another possible limitation is that cases and controls were recruited in 20 major hospitals, but as these hospitals cover 16.8% of the Spanish population aged ≥ 65 years we believe that the study is representative of the older Spanish population. Also, we have not collected information on patients’ influenza-like illness in previous seasons, but previous episodes of influenza does not usually act as a confounding factor that needs to be controlled for in studies evaluating influenza VE [30]. Finally, the low statistical power in the investigation of VE against influenza B virus because of the very low number of cases in the two seasons studied was another limitation.

In conclusion, the results of this study show that influenza vaccination was effective in preventing hospitalisations because of influenza in individuals who are elderly. The point estimates of the adjusted VE were highest in patients without high-risk medical conditions, in patients in the 65–79 years of age group and against the influenza A(H1N1)pdm09 subtype compared with the A(H3N2) subtype, although the 95% confidence limits overlapped. Finally, we found that VE was similar between vaccination only in the current season and vaccination in both the current and the previous seasons.

## Notes

**The members of the Project PI12/02079 Working Group are:***Andalusia:* J.M. Mayoral (Servicio de Vigilancia de Andalucía), J. Díaz-Borrego (Servicio Andaluz de Salud), A. Morillo (Hospital Universitario Virgen del Rocío), M.J. Pérez-Lozano (Hospital Universitario Virgen de Valme), J. Gutiérrez (Hospital Universitario Puerta del Mar), M. Pérez-Ruiz, M.A. Fernández-Sierra (Complejo Hospitalario Universitario de Granada). *Castile and Leon****:*** S. Tamames (Dirección General de Salud Pública, Investigación, Desarrollo e Innovación, Junta de Castilla y León), S. Rojo-Rello (Hospital Clínico Universitario de Valladolid), R. Ortiz de Lejarazu (Universidad de Valladolid), M.I. Fernández-Natal (Complejo Asistencial Universitario de León), T. Fernández-Villa (GIIGAS-Grupo de Investigación en Interacción Gen-Ambiente y Salud, Universidad de León), A. Pueyo (Hospital Universitario de Burgos), Vicente Martin (Universidad de León; CIBERESP). *Catalonia:* A. Vilella (Hospital Clínic), M. Campins, A. Antón (Hospital Universitari Vall d’Hebron, Universitat Autónoma de Barcelona), G. Navarro (Corporació Sanitària i Universitaria Parc Taulí), M. Riera (Hospital Universitari MútuaTerrassa), E. Espejo (Hospital de Terrassa), M.D. Mas, R. Pérez (ALTHAIA, Xarxa Hospitalaria de Manresa), J.A. Cayla, C. Rius (Agència de Salut Pública de Barcelona; CIBERESP), P. Godoy (Agència de Salut Pública de Catalunya; Institut de Recerca Biomèdica de Lleida, Universitat de Lleida; CIBERESP), N. Torner (Agència de Salut Pública de Catalunya; Universitat de Barcelona; CIBERESP), C. Izquierdo, R. Torra (Agència de Salut Pública de Catalunya), L. Force (Hospital de Mataró), A. Domínguez, N. Soldevila, I. Crespo (Universitat de Barcelona; CIBERESP), D. Toledo (Universitat de Barcelona). *Madrid****:*** J. Astray, M.F. Domínguez-Berjon, M.A. Gutiérrez, S. Jiménez, E. Gil, F. Martín, R. Génova-Maleras (Consejería de Sanidad), M.C. Prados, F. Enzzine de Blas, M.A. Salvador, J. Rodríguez, M. Romero (Hospital Universitario la Paz), J.C. Galán, E. Navas, L. Rodríguez (Hospital Ramón y Cajal), C.J. Álvarez, E. Banderas, S. Fernandez (Hospital Universitario 12 de Octubre). *Navarra****:*** J. Chamorro (Complejo Hospitalario de Navarra), I. Casado, J. Díaz (Instituto de Salud Pública de Navarra), J. Castilla (Instituto de Salud Pública, Instituto de Investigación Sanitaria de Navarra; CIBERESP). *The Basque Country****:*** M. Egurrola, M.J. López de Goicoechea (Hospital de Galdakao). *Valencia Community*: M. Morales-Suárez-Varela (Universidad de Valencia; CIBERESP), F. Sanz (Consorci Hospital General Universitari de Valencia).

## References

[1] Treanor JJ. Influenza Viruses Including Avian Influenza. In: Bennet JE, Dolin R, Blaser MJ, editors. Mandell, Douglas, and Bennett's Principles and Practice of Infectious Diseases. 8th ed. Philadelphia: Elsevier; 2015. p. 2000-29.

[2] World Health Organization (WHO). Influenza (Seasonal): Fact sheet. Geneva: WHO; Nov 2016. [Accessed 2 Aug 2016]. Available from: http://www.who.int/mediacentre/factsheets/fs211/en/

[3] Foppa IM Cheng PY Reynolds SB Shay DK Carias C Bresee JS Deaths averted by influenza vaccination in the U.S. during the seasons 2005/06 through 2013/14. Vaccine. 2015;33(26):3003-9. 10.1016/j.vaccine.2015.02.042 25812842 PMC4834450

[4] Bonmarin I Belchior E Lévy-Bruhl D Impact of influenza vaccination on mortality in the French elderly population during the 2000-2009 period. Vaccine. 2015;33(9):1099-101. 10.1016/j.vaccine.2015.01.023 25604800

[5] Lang PO Mendes A Socquet J Assir N Govind S Aspinall R Effectiveness of influenza vaccine in aging and older adults: comprehensive analysis of the evidence. Clin Interv Aging. 2012;7:55-64. 10.2147/CIA.S25215 22393283 PMC3292388

[6] Beyer WE McElhaney J Smith DJ Monto AS Nguyen-Van-Tam JS Osterhaus AD Cochrane re-arranged: support for policies to vaccinate elderly people against influenza. Vaccine. 2013;31(50):6030-3. 10.1016/j.vaccine.2013.09.063 24095882

[7] Sistema de Vigilancia de la Gripe en España. Informe de Vigilancia de la Gripe en España. Temporada 2013-2014 (Desde la semana 40/2013 hasta la semana 20/2014). [Influenza surveillance report in Spain. Season 2013/2014. (From week 40/2013 until week 20/2014)]. Madrid: Instituto de Salud Carlos III. [Accessed 10 Nov 2016]. Spanish. Available from: http://www.isciii.es/ISCIII/es/contenidos/fd-servicios-cientifico-tecnicos/fd-vigilancias-alertas/fd-enfermedades/Informe_Vigilancia_GRIPE_2013-2014_v19122014.pdf

[8] Sistema de Vigilancia de la Gripe en España. Informe de Vigilancia de la Gripe en España. Temporada 2014-2015 (Desde la semana 40/2014 hasta la semana 20/2015). [Influenza surveillance report in Spain. Season 2014/2015. (From week 40/2014 until week 20/2015)]. Madrid: Instituto de Salud Carlos III. [Accessed 22 Aug 2016]. Spanish. Available from: http://www.isciii.es/ISCIII/es/contenidos/fd-servicios-cientifico-tecnicos/fd-vigilancias-alertas/fd-enfermedades/pdf_2015/Informe_Vigilancia_GRIPE_2014-2015_vf_29092015.pdf

[9] Fiore AE, Bridges CB, Katz JM, Cox NJ. Inactivated influenza vaccines. In: Plotkin SA, Orenstein WA, Offit PA, editors. Vaccines. 6th ed. Philadelphia: Elsevier; 2013. p. 257-93.

[10] R Core Team. R: A Language and Environment for Statistical Computing. Vienna, Austria: R Foundation for Statistical Computing. [Accessed 1 Aug 2017]. Available from: https://www.R-project.org

[11] Remschmidt C Rieck T Bödeker B Wichmann O Application of the screening method to monitor influenza vaccine effectiveness among the elderly in Germany. BMC Infect Dis. 2015;15(1):137. 10.1186/s12879-015-0882-3 25887460 PMC4371628

[12] Talbot HK Griffin MR Chen Q Zhu Y Williams JV Edwards KM Effectiveness of seasonal vaccine in preventing confirmed influenza-associated hospitalizations in community dwelling older adults. J Infect Dis. 2011;203(4):500-8. 10.1093/infdis/jiq076 21220776 PMC3071231

[13] Minodier L Blanchon T Souty C Turbelin C Leccia F Varesi L Influenza vaccine effectiveness: best practice and current limitations of the screening method and their implications for the clinic. Expert Rev Vaccines. 2014;13(8):1039-48. 10.1586/14760584.2014.930666 24946796

[14] Puig-Barberà J Mira-Iglesias A Tortajada-Girbes M Lopez-Labrador FX Belenguer-Varea A Carballido-Fernandez M Effectiveness of influenza vaccination programme in preventing hospital admissions, Valencia, 2014/15 early results. Euro Surveill. 2015;20(8):21044. 10.2807/1560-7917.ES2015.20.8.21044 25742432

[15] Pierse N Kelly H Thompson MG Bissielo A Radke S Huang QS Influenza vaccine effectiveness for hospital and community patients using control groups with and without non-influenza respiratory viruses detected, Auckland, New Zealand 2014. Vaccine. 2016;34(4):503-9. 10.1016/j.vaccine.2015.11.073 26685091

[16] Petrie JG Ohmit SE Cheng CK Martin ET Malosh RE Lauring AS Influenza vaccine effectiveness against antigenically drifted influenza higher than expected in hospitalized adults: 2014-2015. Clin Infect Dis. 2016;63(8):1017-25. 10.1093/cid/ciw432 27369320

[17] Qin Y Zhang Y Wu P Feng S Zheng J Yang P Influenza vaccine effectiveness in preventing hospitalization among Beijing residents in China, 2013-15. Vaccine. 2016;34(20):2329-33. 10.1016/j.vaccine.2016.03.068 27026147

[18] Turner N Pierse N Huang QS Radke S Bissielo A Thompson MG Interim estimates of the effectiveness of seasonal trivalent inactivated influenza vaccine in preventing influenza hospitalisations and primary care visits in Auckland, New Zealand, in 2014. Euro Surveill. 2014;19(42):20934. 10.2807/1560-7917.ES2014.19.42.20934 25358042

[19] McLean HQ Thompson MG Sundaram ME Kieke BA Gaglani M Murthy K Influenza vaccine effectiveness in the United States during 2012-2013: variable protection by age and virus type. J Infect Dis. 2015;211(10):1529-40. 10.1093/infdis/jiu647 25406334 PMC4407759

[20] McElhaney JE Zhou X Talbot HK Soethout E Bleackley RC Granville DJ The unmet need in the elderly: how immunosenescence, CMV infection, co-morbidities and frailty are a challenge for the development of more effective influenza vaccines. Vaccine. 2012;30(12):2060-7. 10.1016/j.vaccine.2012.01.015 22289511 PMC3345132

[21] Spadea A Unim B Colamesta V Meneghini A D’Amici AM Giudiceandrea B Is the adjuvanted influenza vaccine more effective than the trivalent inactivated vaccine in the elderly population? Results of a case-control study. Vaccine. 2014;32(41):5290-4. 10.1016/j.vaccine.2014.07.077 25087677

[22] Skowronski DM Chambers C Sabaiduc S De Serres G Winter AL Dickinson JA A perfect storm: impact of genomic variation and serial vaccination on low influenza vaccine effectiveness during the 2014-2015 season. Clin Infect Dis. 2016;63(1):21-32. 10.1093/cid/ciw176 27025838 PMC4901864

[23] Treanor JJ. Influenza Viruses. In: Kaslow RA, Stanberry LR, Le Duc JW, editors. Viral Infections of Humans. 5th ed. New York: Springer; 2014. p. 455-78.

[24] High K Immunizations in older adults. Clin Geriatr Med. 2007;23(3):669-85, viii-ix. 10.1016/j.cger.2007.03.007 17631240

[25] Flannery B Clippard J Zimmerman RK Nowalk MP Jackson ML Jackson LA Early estimates of seasonal influenza vaccine effectiveness - United States, January 2015. MMWR Morb Mortal Wkly Rep. 2015;64(1):10-5. 25590680 PMC4584793

[26] Castilla J Martínez-Baz I Navascués A Fernandez-Alonso M Reina G Guevara M Vaccine effectiveness in preventing laboratory-confirmed influenza in Navarre, Spain: 2013/14 mid-season analysis. Euro Surveill. 2014;19(6):20700. 10.2807/1560-7917.ES2014.19.6.20700 24556347

[27] World Health Organization Recommended composition of influenza virus vaccines for use in the 2015–2016 northern hemisphere influenza season. Wkly Epidemiol Rec. 2015;90(11):97-108. 25771542

[28] Castilla J Navascués A Fernández-Alonso M Reina G Pozo F Casado I Effectiveness of subunit influenza vaccination in the 2014-2015 season and residual effect of split vaccination in previous seasons. Vaccine. 2016;34(11):1350-7. 10.1016/j.vaccine.2016.01.054 26854911

[29] Neuzil KM How can we solve the enigma of influenza vaccine-induced protection? J Infect Dis. 2015;211(10):1517-8. 10.1093/infdis/jiu651 25416811 PMC4407760

[30] Castilla J Navascués A Fernández-Alonso M Reina G Albéniz E Pozo F Effects of previous episodes of influenza and vaccination in preventing laboratory-confirmed influenza in Navarre, Spain, 2013/14 season. Euro Surveill. 2016;20(22):30243. 10.2807/1560-7917.ES.2016.21.22.30243 27277013

